# Threshold Dependence of Deep- and Near-subwavelength Ripples Formation on Natural MoS_2_ Induced by Femtosecond Laser

**DOI:** 10.1038/srep19571

**Published:** 2016-01-22

**Authors:** Yusong Pan, Ming Yang, Yumei Li, Zhenhua Wang, Chunling Zhang, Ying Zhao, Jianghong Yao, Qiang Wu, Jingjun Xu

**Affiliations:** 1 The MOE Key Laboratory of Weak Light Nonlinear Photonics, TEDA Applied Physics Institute and School of Physics, Nankai University, Tianjin 300457, China; 2 The Institute of Photoelectronic Thin Film Devices and Technology, Nankai University, Tianjin 300071, China

## Abstract

Deep sub-wavelength ripples (DSRs) and near sub-wavelength ripples (NSRs) with uniform periods of ~160 nm and ~660 nm generated at the MoS_2_-vacuum interface is reported for the first time by the processing of femtosecond laser (800 nm, 120 fs, 1 kHz) in this paper. The DSRs and NSRs formation fluence thresholds are experimentally determined as 160 mJ/cm^2^ and 192 mJ/cm^2^, respectively. In addition, the ripple period is insensitive to the pulse number. Moreover, Raman analyses show that the MoS_2_ lattice in the irradiated area does not exhibit oxidation at room environment and the crystalline representation is well preserved in NSRs region. We attribute our result to the joint interactions of the spallation and sublimation of layered MoS_2_ together with the laser induced surface plasmon polaritons and propose an explanation to the threshold dependence of the ripple period. Our study provides some insights for ultrafast laser-matter interactions and indicates a simple effective method for future nano-fabrication of MoS_2_.

A universal phenomenon in the field of femtosecond (fs) laser ablation is the appearance of periodic ripples on the material surface with a period smaller than the laser wavelength and laser polarization dependence^1^. The formation of this kind of nanostructure has been motivating quite a few studies in the past decade not only because it is a special way to understand the fundamental physics of fs laser-matter interactions^2^,^3^,^4^,^5^,^6^,^7^, but also because it is potentially applicable in nano-science and technology. This kind of sub-wavelength patterns has shown its capabilities to alter optical properties of materials^8^, fabricate SERS substrates^9^, conduct large area lithography^10^ and realize high efficient light collection in a broad wavelength range^11^. To date, lots of experiments are performed on semiconductors^2^,^5^,^12^, metals^8^,^10^ and dielectrics^7^,^13^, but the materials with graphite-like structure are seldom involved.

Recently, molybdenum disulfide (MoS_2_), a two-dimensional (2D) semiconductor material with strong bonding (mainly covalent) within layers and weak interatomic interactions (van der Waals forces) between their layered structures, has attracted great research interests because of its unique structure related electronic and optical properties^14^,^15^,^16^ and promising applications such as double-layer capacitor^17^, transistors^18^,^19^, photo detectors^20^ and photovoltaic devices^21^ that substantially extends the range of possible nanostructures and devices for nano-fabrication, as well as its up to date nono-fabrication studies via laser^22^,^23^. Besides, MoS_2_ is chemically stable in dry oxygen environment and only when heated up to 300 °C does molybdenum trioxide form^24^, which makes it a durable material. These excellent properties determine that MoS_2_ is promising in photoelectric field and in the meanwhile can be easily carved with low tendency of oxidation.

In this paper, we report for the first time, on the discovery of fs laser-induced periodic surface ripples on MoS_2_. Uniform DSRs and NSRs with the respective period of ~160 nm and ~660 nm are produced by the fs laser (800 nm, 120 fs, 1 kHz) processing on natural MoS_2_. The ripple period is insensitive to the pulse number and the formation thresholds for the DSRs and NSRs are experimentally determined as 160 mJ/cm^2^ and 192 mJ/cm^2^, respectively. Then micro-Raman spectral analysis is performed to detect the MoS_2_ lattice structural changes in the irradiated area. We find MoS_2_ does not suffer from oxidation and its crystalline representation is well preserved after laser irradiation. These results are ascribed to the sublimation and spallation of MoS_2_ during the fs laser processing, in which the sublimated and spalled material takes away redundant laser energy together with the noncrystalline part meanwhile inhibits the melting effect. Finally, an explanation is proposed for the threshold dependence of ripple period. Our study indicates a promising technique for future nano-fabrication on MoS_2_ and provide a fundamental understanding for the fs laser-matter interactions of 2D materials.

## Results

**Figure 1:**
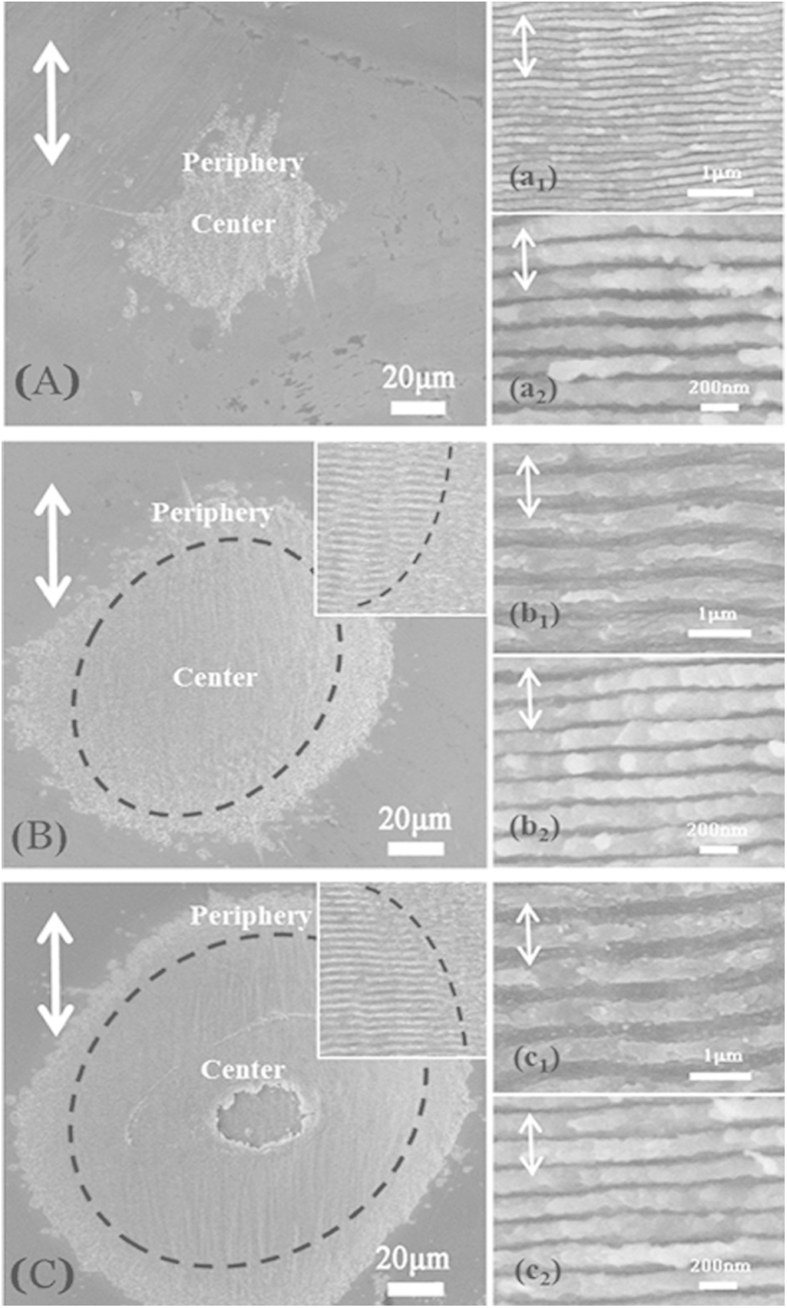
SEM images of MoS_2_ surface irradiated by fs laser beam with 150 pulses for 3 different incident fluences in vacuum: (**A**) 90 mJ/cm^2^, (**B**) 180 mJ/cm^2^ and (**C**) 270 mJ/cm^2^. The details in their central and the periphery of the structured areas are illustrated by (a_1_–c_1_) and (a_2_–c_2_), respectively. The double arrows in the figure indicate the polarization directions of the incident laser. Black dashed line stands for the boundary between NSRs and DSRs. Insets in (**B**,**C**) are details near the boundary between the NSRs and the DSRs.

Figure 1 depicts the scanning electron microscopy (SEM) images of MoS_2_ surface irradiated by fs laser beam of different incident fluences with 150 pulses. A damage spot with an irradiation fluence of 90 mJ/cm^2^ is shown in Fig. 1(A). The generated ripples represent a period of approximately 160 nm which is significantly smaller than the laser wavelength <*λ*/2, so we just name them DSRs. The energy fluence hit on the spot in this condition is slightly above the ripple formation threshold of bulk MoS_2_, given that in the case of 80 mJ/cm^2^ or less, no structures are observed by the SEM. The coverage of DSRs with an orientation perpendicular to the laser polarization are detected both in the center and the periphery of the damage spot, as shown in Fig. 1(a1,a2). The ripples represent rows of wide fluctuant ridges and narrow dark grooves, and few re-deposited fragments are found on the irradiation area.

When the irradiation fluence is increased to 180 mJ/cm^2^, the average period turns into about 660 nm in the central ablation region as marked out in Fig. 1(B), where quite regular and continuous ripples with relatively wider grooves emerge. Due to the ~0.8 *λ* spatial distance being quite near the laser wavelength, we name ripples of this kind the NSRs. While we also find the ripple period in the periphery of the damage part remains unchanged (~160 nm), compared Fig. 1(a2,b2). Moreover, from the inset of Fig. 1(B), we can see the ripple period on MoS_2_ displays an abrupt change from NSR to DSR for this case.

At a higher fluence of 270 mJ/cm^2^, it can be seen that further increase in laser fluence doesn’t change the ripple morphology in the central ablation region, as demonstrated in Fig. 1(C). Again, a saltation of ripple period from about 660 nm to 160 nm can be observed (see the inset of Fig. 1(C)). In spite of the considerable removals of the upper layers of MoS_2_, the ripples are still formed at the bottom of the small crater and they exhibit a period of approximately 660 nm as well (see Fig. 1(c1)).

**Figure 2:**
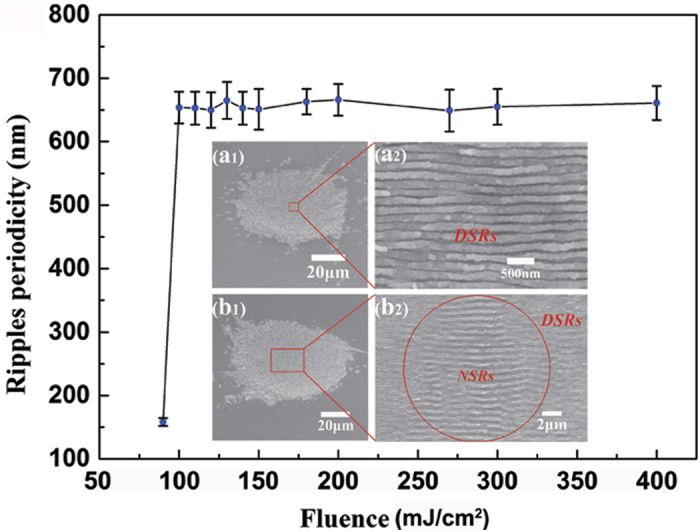
Central ripple periodicity of MoS_2_ induced by fs laser with a pulse number of 150 and incident fluences from 90 mJ/cm^2^ to 400 mJ/cm^2^. Insets are SEM images that depict the cases of 90 mJ/cm^2^ (a_1_,a_2_) and 100 mJ/cm^2^ (b_1_,b_2_), between which the DSR and NSR transition fluence of incident beam lies.

Figure 2 represents the evolution of ripple periods as the incident fluence increases with the number of pulses fixed to 150, where both the DSRs and NSRs formation conditions can be separated. We can see that the ripples do not emerge until the laser fluence goes up to about 90 mJ/cm^2^ and they display a sole period of approximately 160 nm. When the fluence is increased to 100 mJ/cm^2^, the NSRs with a period of about 660 nm emerge in the central of the ablation area. After that, the near sub-wavelength period in the center hardly changes with the increasing laser fluence. The appearance of Fig. 2 clearly indicates that the DSRs and NSRs each has a formation threshold fluence.

**Figure 3:**
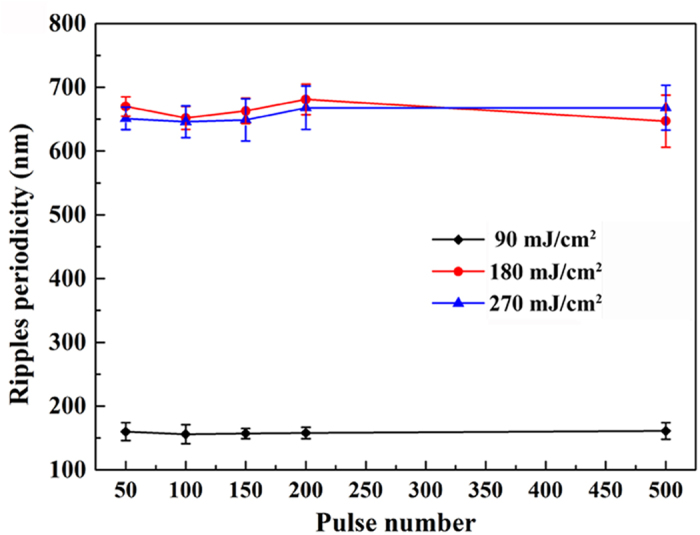
Surface ripple periods in central ablation region measured for different pulse numbers with incident laser fluences of 90 mJ/cm^2^, 180 mJ/cm^2^ and 270 mJ/cm^2^, respectively.

Previous studies of fs laser induced periodic surface structures on other semiconductors^2^,^25^ demonstrate that the ripple period decreases monotonously with the increase of pulse number, which is not quite consistent with our investigation of MoS_2_, at least in our experimental range. Figure 3 shows the evolution of ripples with increasing pulse numbers in the central ablation region. The diamonds, spots and triangles in the graph each represent a fixed irradiation fluence of 90 mJ/cm^2^, 180 mJ/cm^2^ and 270 mJ/cm^2^ respectively, indicating that the periods of both the DSRs and the NSRs keep almost constant regardless of the number of laser pulses.

**Figure 4:**
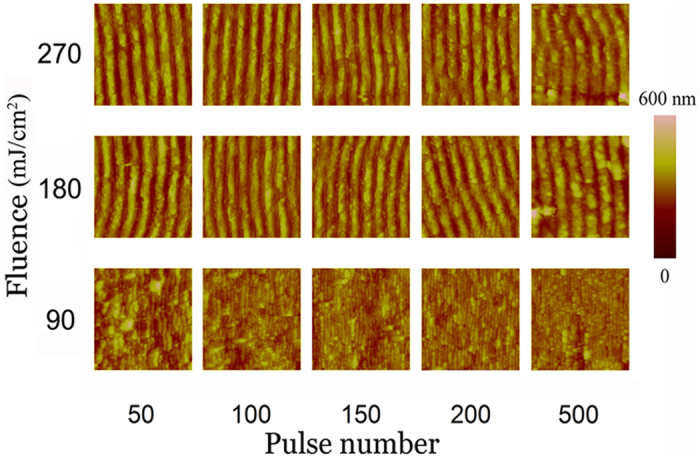
AFM images for the cases of various pulse numbers and incident fluences. Each of them demonstrate 5 × 5 *μ*m area.

As the height of the ripples is also a critical concern for nano-fabrication, we study the ripple height change with variations of pulse number and incident fluence by the atomic force microscope (AFM). Figure 4 depicts a series of 5 × 5 *μ*m area AFM images of MoS_2_ surface irradiated by fs laser beam with 50, 100, 150, 200 and 500 pulses, respectively for three different incident fluences (90 mJ/cm^2^, 180 mJ/cm^2^ and 270 mJ/cm^2^).

**Figure 5:**
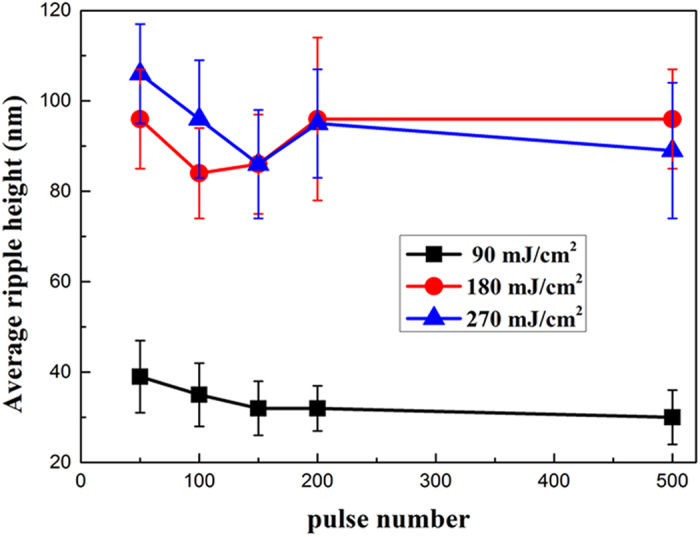
Average ripple height measured according to Fig. 4 for three different fluences with various pulse numbers.

The information of the ripple height are analysed from each AFM image and their average heights versus the pulse number are obtained, as shown in Fig. 5. We can see the average ripple heights of both the DSRs and the NSRs show a slight drop at first and then the heights become relatively stable as the pulse number is larger than about 200. The experimental result indicate the stablized average ripple heights of the DSRs and the NSRs to be ~30 nm and ~90 nm, respectively, which is good news for the nano-etching of fs laser.

**Figure 6:**
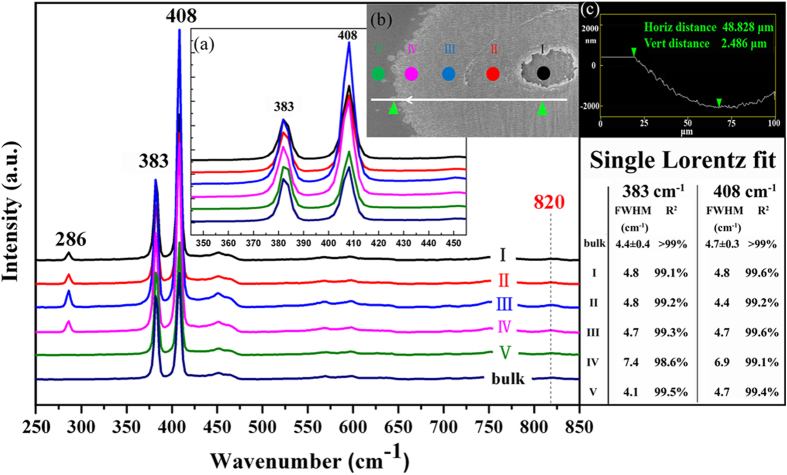
Micro-Raman spectra (514.5 nm excitation) of a sample that is treated by fs laser with a fluence of 270 mJ/cm^2^ and a pulse number of 150 in positions ranging from the center to the periphery of the damage spot. The spectrum measured for bulk MoS_2_ is used for comparison. The FWHM of the two main Raman peaks are well fitted by single Lorentz function (with coefficient of determination R^2^ > 0.99) shown in the right tabulation. The inset (**a**) highlights two main Raman peaks corresponding to 383 cm^−1^ and 408 cm^−1^. The inset (**b**) shows the SEM image of the sample. The coloured dots indicate the tested regions that correspond to lines I-V. Inset (**c**) is the height information along the white line of inset (**b**), where 48.828 *μ*m and 2.486 *μ*m are the horizontal and vertical distance between the two green triangles, respectively.

In order to further characterize the structure of the irradiated area, micro-Raman spectral analysis are performed in regions varied from the ablation center to the periphery of the damage district that cover regions of the NSRs (line I, II, III), the DSRs (line IV) and the non-structured but irradiated part (line V), as indicated in Fig. 6 and its inset (b). After being processed by fs laser, the major difference between the laser structured and the non-structured part is the additional 286 cm^−1^ peak. While the other two peaks centered at 383 cm^−1^ and 408 cm^−1^ which are the characteristic first-order Raman peaks of crystalline MoS_2_ remained surprisingly untouched, except for the peak broadenings of the DSRs region shown in the right tabulation where the FWHM of the two main Raman peaks are well fitted by single Lorentz function with coefficient of determination R^2^ > 0.99.

## Discussion

**Figure 7:**
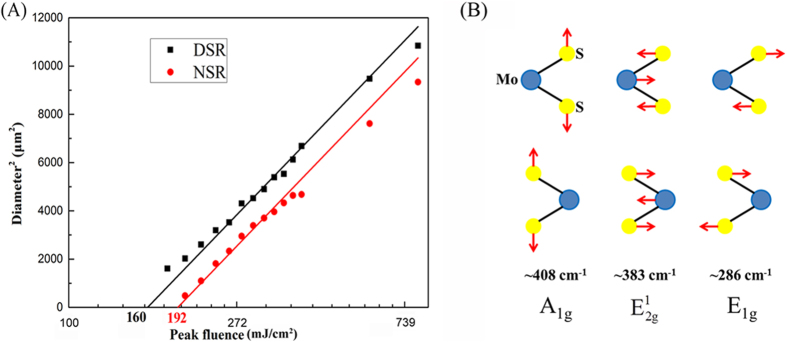
(**A**) Squared diameter of the structured areas (squares belong to the DSR region and circles belong to the NSR region) on MoS_2_ surface versus the incident peak fluence of the laser pulse. Solid lines are linear regressions within the semi-logarithmic plot. (**B**) Three schematic first-order Raman active modes at ~408 cm^−1^ (A_1*g*_), ~383 cm^−1^ (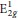) and ~286 cm^−1^ (E_1*g*_) of bulk MoS_2_.

Note that the fluences we mentioned previously are the average incident fluences that equal to the energy per laser pulse divided by the focused Gaussian beam area on MoS_2_, which cannot stand for the real fluences received by the structured regions, since the focused beams have larger areas. To find the actual threshold fluences, we measure the diameters of the DSR and NSR regions versus various fluences and extrapolate down to zero^26^, as represented in Fig. 7(A) where the square of diameters are depicted versus increasing peak fluence of the laser pulses. Extending the regressions of these lines to zero yields the actual threshold values of DSRs and NSRs to be about 160 mJ/cm^2^ and 192 mJ/cm^2^, respectively. That is why we could not observe rippled structure at the average incident fluence of 80 mJ/cm^2^ (with its peak fluence of 160 mJ/cm^2^ that is not enough for the observation of DSRs) or less and we did not see the NSRs until the average incident fluence increases to 100 mJ/cm^2^ (with its peak fluence of 200 mJ/cm^2^ that is larger than the NSRs formation threshold).

Viewed from the Raman spectra above, the 383 cm^−1^ peak is associated with the in-plane opposite motion between two S atoms and Mo atom and the 408 cm^−1^ peak is attributed to the out-of-plane vibration of S atoms in opposite directions, while the peak centered at 286 cm^−1^ can result from either the in-plane opposite vibration of the two S atoms which also belongs to first-order Raman peaks of crystalline MoS_2_ (see Fig. 7(B)) or the characteristic peak of MoO_3_. We exclude the later for the absence of the 820 cm^−1^ peak which is a signature of oxidation of MoS_2_ to MoO_3_^27^. Usually the 286 cm^−1^ peak should be prohibited in Raman back-scattering experiment on the basal plane of MoS_2_ crystal^28^, since Raman scattering is inelastic scattering caused by movements of molecules and its intensity is determined by the polarization of incident and scattered light plus change in polarizibility, i.e.


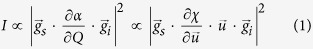


where 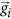 and 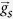 denote the polarization vectors of incident and scattered light, *α* and *χ* are the polarizability and electric susceptibility, Q and 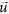 denote the canonical coordinate and molecular displacement, respectively. We introduce the notation (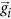, 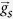) and define a rectangular coordinate system with the x-y plane parallel to the MoS_2_ layer planes and the z axis parallel to the c axis to describe the scattering geometries. For the incoming light along the c axis, the A_1*g*_ mode can be detected in (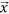, 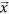), (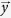, 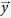) and (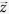, 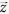) scattering geometries and the 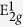 mode can be detected in (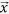, 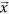), (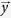, 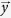) and (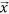, 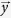) scattering geometries. While in contrast, the E_1*g*_ mode demands for a scattering geometry involving a z-component, namely, (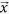, 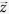), (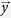, 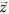) and (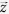, 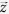)^29^. However, in the fs laser processed area, due to the tilt of the broken surface layers along the curved outline of the ripples from the original basal plane, the same incoming light is no longer parallel to the c axis of the surface MoS_2_ layers but equivalent to oblique incidence, thus making z-component polarization of the scattered light be collected by the Raman back-scattering system much easier. So we speculate the tilt of the layered plane along the ripple outline to be the cause of the emergence of the 286 cm^−1^ peak.

These Raman spectra reveal that MoS_2_ kept unoxidized at room environment, regardless of the surface nano-structure. Furthermore, the inset (a) and (c) in Fig. 6 show in detail the widths of characteristic peaks (383 cm^−1^ and 408 cm^−1^), where little broadening and shifting (except for line IV) indicate that no distortion of short-range structures and bond angles in the lattice appears, which means that no amorphous, poly-crystalline or nano-crystalline is left on the surface after the laser processing. While the peak broadenings of line IV indicate an imperfect crystallization of MoS_2_ and mainly amorphous representation. Such a transition between the two phases after irradiation results from two different solidification processes: amorphization and re-crystallization. Their difference is ascribed to the amount of energy deposited in the semiconductor (the temperature) and the consequent solidification speed and the amorphous phase forms when solidification speed is higher than a critical value^30^,^31^,^32^. So in the DSRs region with lower temperatures, the MoS_2_ has not enough time to re-crystallize from the melt, leaving part of the material in an amorphous state.

The Raman analysis indicate that the crystalline representation of MoS_2_ counter-intuitively survives the fs laser etching in the NSRs region, which is different from the cases of traditional semiconductor with surfaces forming amorphous or polymorphic phases for all the structured part after nonequilibrium melting of the surface^32^,^33^. At fluences above NSRs formation, the lattice temperature of the irradiation part can reach a few thousand Kelvins within a few picoseconds^5^,^6^ that well exceeds the temperature for sublimation of MoS_2_ (723 K) and it is easy for the weak interlayer van der Waals bonds to release. Therefore, we believe the sublimation and easy spallation property of MoS_2_ plays an important role during the etching process, for the removal of material fragments in the NSRs region promotes the elimination of non-crystalline exhibition and results in no amorphous or polymorphic MoS_2_.

The sublimation together with spallation and fragmentation of MoS_2_ is also beneficial to keep the regularity of surface structures. The rippled pattern does not deform or efface after laser processing and the supposed erasure of surface patterns caused by ultrafast melting^5^ is suppressed. Even in the cases where the incident fluences are much higher than the NSRs formation threshold, layered MoS_2_ are violently ablated away meanwhile take away a lot of excess energy leaving a crater (see in Fig. 1(C)), as a result, regular ripples can still be generated beneath the ablated surface layers. This explains the well preserved ripple patterns with various incident fluences and pulse numbers in our experiments.

During the ultrafast and strong processing of fs laser the irradiated region is pushed into an extreme non-equilibrium state, our fs laser induced periodic ripples cannot be described by the simplified classical model, which educes that ripple distance should be equal to *λ* in normal incidence^2^. Once a sufficiently large amount of electrons is excited from the valance band by our fs laser, MoS_2_ can transiently turn from a semiconducting to a metal-like state well before the thermal relaxations of free carriers, leading to the changes of optical properties (e.g. the dielectric permittivity) and the production of surface plasma polaritons (SPPs) whose wavelength lie below the incident light according to dispersion relation^34^. So we attribute our nanoscale periodicity to the excitation of SPPs which is caused by the coherent interaction of the incident field with free electrons created from MoS_2_ and the complex dielectric permittivity of the highly excited surface can be described using the Drude model expressed as


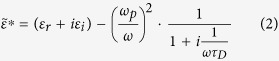


where *ε*_*r*_ and *ε*_*i*_ are the real and imaginary part of dielectric constant of the unexcited MoS_2_, *ω* and *τ*_*D*_ denote the angular frequency of the incident laser wave and the Drude damping time (*τ*_*D*_ = 90 fs^35^). *ω*_*p*_ stands for the plasma frequency given as *ω*_*p*_ = (*e*^2^N_*e*_/m_*opt*_m_0_*ε*_0_)^1/2^, [*e*: electron charge, N_*e*_: density of excited carriers, 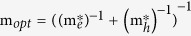 with 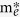 and 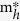 denoting the mobility effective masses of electrons and holes, m_0_: free electron mass, *ε*_0_: vacuum dielectric permittivity]. Band filling and band gap renormalization effects are neglected here. Then the wavelength of SPPs at the metal-dielectric interface according to dispersion relation is expressed as


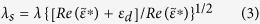


where *λ* = 800 nm, *ε*_*d*_ = 1 is dielectric constant of the vacuum. Based on the SPPs-laser interference model^2^, the ripple period is calculated by Λ = *λ*(sin*θ* ± *λ*/*λ*_*s*_)^−1^ with *θ* being the laser incident angle (*θ* = 0, Λ = *λ*_*s*_ for normal incidence).

On the basis of our theory and by adopting the values of 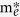 and 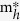 to be ~0.61m_0_^35^ and *ε*_*r*_ = 8.5 for the direction along the c axis^36^, we estimate the fs laser excited transient carrier density to be about 5.08 × 10^21^ cm^−3^ for Λ = 160 nm and 6.19 × 10^21^ cm^−3^ for Λ = 660 nm, both of which exceed the critical value of 5.05 × 10^21^ cm^−3^ (with Re (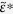) = −1) above which the SPPs can be produced.

According to Eq. (2) and Eq. (3), the ripple distance should have varied continuously with the carrier density. However, we experimentally obtain ripples with only two separate periods, which means there are other physical factors involving in. Besides, the threshold fluence has an increment of 20% from 160 mJ/cm^2^ to 192 mJ/cm^2^ while the corresponding carrier density is increased by 21.9% from 5.08 × 10^21^ cm^−3^ to 6.19 × 10^21^ cm^−3^, which implies that the effective two photon absorption coefficient for 160 mJ/cm^2^ fluence is abnormally larger than that of 192 mJ/cm^2^ fluence (see in methods). Clearly, the extreme conditions created by fs laser-matter interactions make interpretation of the exact physical mechanism a difficult task. One important character of 2D MoS_2_ is the saturable absorption of light^37^ mainly arises from the limited valance band electrons, which should not be neglected. The formation thershold intensities of DSRs (160 mJ/cm^2^ corresponding to ~1330 GW/cm^2^) and NSRs (192 mJ/cm^2^ corresponding to ~1600 GW/cm^2^) both well exceed the reported MoS_2_ ultrafast saturation intensity of about 400 ~ 800 GW/cm^2^ that indicate the occurrence of the saturated absorptions^38^.

These facts induce us to propose our scenario here. At lower fluence (below the DSRs formation region), the excited carriers have insufficient time to decay back within the fs pulse and the frequency and wavelength of produced plasma vary as a function of the carrier density determined by the incident fluence. As the laser fluence increases, the light field does not stop redistributing the carriers until it reaches ~160 mJ/cm^2^ when the valance band starts to fail to offer more electrons for excitation, making the density of free carriers become saturated. Then the carrier density no longer increases with the laser fluence and so does the SPPs wavelength *λ*_*s*_ and ripple period Λ. At higher fluence (at and above ~192 mJ/cm^2^ the NSRs formation region), the laser field is strong enough to break the intralayer covalent bonds, causing another amount of electrons releasing from the MoS_2_ before the molecular units decompose and flee from the surface. Thus a sudden increase of the carriers occurs above a critical fluence, leading to the observation of a sudden transition of Λ from ~160 nm to ~660 nm in our experiments. So actually, our result does not violate the previous mechanism of subwavelength structures formed by SPPs.

Finally, it is worthwhile to mention that the properties of MoS_2_ change notably when the thickness is within a few molecular layers due to structural related quantum effects^14^, which implies difference in behaviour of sufficient thin samples from the bulk. The fact that the bandgap broadens as the number of layer decreases may lead to variations of threshold fluences and ripple periods, since broader bandgap makes harder phonton absorption and thus a reduction of the excited carriers for the same incident fluence and the role of substrate may also not be neglected (for example, fs laser induced subwavelength ripples on SiO_2_ have a direction parallel to polarization^7^), which requires large-area, high-quality thin samples for systematic further study to precisely know the thickness dependence. In addition, our simple effective method (one irradiated spot with many ripple lines) for fabricating small regular structures could lead to anisotropy to physical properties of the in-plane symmetric 2D MoS_2_ with the directions along and perpendicular to the ripple lines and related function modifications, as very recently achieved in transition-metal oxides^39^. This promising application issue is a subject to further explorations.

In summary, we have produced uniform periodic surface ripples on natural MoS_2_ by irradiation with linearly polarized fs laser pulses in vacuum. The threshold fluences to generate DSRs (~160 nm) and NSRs (~660 nm) are experimentally determined as 160 mJ/cm^2^ and 192 mJ/cm^2^, respectively. Besides, the ripple period is insensitive to the pulse number. Furthermore, Raman spectra indicate that most of the irradiated area (the NSRs region) keeps crystalline and does not exhibit amorphous or oxidation. These phenomena are given rise to the sublimation and spallation of MoS_2_ during the fs laser processing, in which the sublimated and spalled material takes away redundant laser energy and noncrystalline part, meanwhile inhibits the ripple erasure caused by melting. Finally, we propose our explanation for the threshold dependence of ripple period. Our study can offer some insights for understanding the physics of self-assembled surface structures of 2D materials induced by fs lasers and indicates a convenient technique for future nano-fabrication and feature modulation on MoS_2_.

## Methods

Our sample is a piece of natural (2H) molybdenum disulfide (SPI supplies, 429 MS-AB) with dimensions of 8 × 8 × 1 mm^3^. A Ti: sapphire-based regenerative laser amplifier system, which generates a linearly-polarized, 800 nm central wavelength beam, with pulse duration of 120 fs and a repetition rate of 1 kHz, was used to perform surface ablation spots. The sample was installed on a controllable X-Y-Z translation stage in a sealed vacuum chamber with its surface locating 10 mm before the focus of a 500 mm focal-length lens and perpendicular to the incident beam. With the help of a mechanical vacuum pump, we were able to obtain a gas pressure of as low as 1 Pa. To eliminate the oxygen thoroughly, we filled in the chamber with nitrogen to 1 atm after the air in the chamber was drawn to about 1 Pa and then started the pump again until the pressure in the chamber reached about 1 Pa. A half-wave plate and a Glan-Taylor prism were arranged to adjust the polarization direction and laser pulse energy in the range of 0 ~ 300 *μ*J. The Gaussian diameter of the laser spot on the MoS_2_ surface was about 170 *μ*m. The number of pulses delivered onto our sample surface (50, 100, 150, 200 and 500 pulses, separately) was controlled by a Pockels cell. The ablation spots on the sample were analyzed by scanning electron microscopy and micro-Raman spectroscopy (514.5 nm laser excitation, with a resolution of ~1.9 cm^−1^).

With the calculated carrier density N, the effective two photon absorption coefficient *β* can be estimated by^3^ 
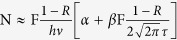
, where F is the fluence, R and *α* ≈ 2 × 10^3^ cm^−1^ for 800 nm light^40^ are the surface reflectivity and linear absorption coefficient, respectively. h is the Planck constant, *ν* is light frequency and *τ* denotes the pulse duration.

## Additional Information

**How to cite this article**: Pan, Y. *et al.* Threshold Dependence of Deep- and Near-subwavelength Ripples Formation on Natural MoS_2_ Induced by Femtosecond Laser. *Sci. Rep.* **6**, 19571; doi: 10.1038/srep19571 (2016).
